# Using Finite Element and Eigenmode Expansion Methods to Investigate the Periodic and Spectral Characteristic of Superstructure Fiber Bragg Gratings

**DOI:** 10.3390/s16020192

**Published:** 2016-02-04

**Authors:** Yue-Jing He, Wei-Chih Hung, Zhe-Ping Lai

**Affiliations:** Department of Electronic Engineering, National Chin-Yi University of Technology, No.57, Section 2, Zhongshan Road, Taiping District, Taichung 41170, Taiwan; roger1427@yahoo.com.tw (W.-C.H.); h50908y@yahoo.com.tw (Z.-P.L.)

**Keywords:** superstructure fiber bragg grating, finite element method, eigenmode expansion method, perfectly matched layer, perfectly reflecting boundary, object meshing method, boundary meshing method

## Abstract

In this study, a numerical simulation method was employed to investigate and analyze superstructure fiber Bragg gratings (SFBGs) with five duty cycles (50%, 33.33%, 14.28%, 12.5%, and 10%). This study focuses on demonstrating the relevance between design period and spectral characteristics of SFBGs (in the form of graphics) for SFBGs of all duty cycles. Compared with complicated and hard-to-learn conventional coupled-mode theory, the result of the present study may assist beginner and expert designers in understanding the basic application aspects, optical characteristics, and design techniques of SFBGs, thereby indirectly lowering the physical concepts and mathematical skills required for entering the design field. To effectively improve the accuracy of overall computational performance and numerical calculations and to shorten the gap between simulation results and actual production, this study integrated a perfectly matched layer (PML), perfectly reflecting boundary (PRB), object meshing method (OMM), and boundary meshing method (BMM) into the finite element method (FEM) and eigenmode expansion method (EEM). The integrated method enables designers to easily and flexibly design optical fiber communication systems that conform to the specific spectral characteristic by using the simulation data in this paper, which includes bandwidth, number of channels, and band gap size.

## 1. Introduction

The development of optical fiber components has led to rapid advancements in the field of communication technology because of their advantageous features such as compactness, lightweight, high signal-transmission capacity, low energy consumption, and resistance to electromagnetic interference, heat, and corrosion. Therefore, optical fiber components have been widely applied in many detection and sensing studies such as optical fiber communication, engineering detection, and medical equipment. Two types of spectral characteristics are currently available in optical fiber gratings. The first type is short-period fiber grating, which is also known as a fiber Bragg grating (FBG) or reflective fiber grating. This type of grating utilizes disturbance principles to couple the input core mode (HE_11_) to the counter-propagation core mode or cladding mode [1,2,3,4]. The second type is long-period fiber grating (LPG), which is also known as transmission-type fiber grating, couples the input core mode (HE_11_) to the copropagation cladding mode [5,6].

To meet the demands for high-speed communication and high product quality, sufficiently increasing the refractive index is crucial. Refractive index changes are inseparable from the photosensitive characteristics of optical fiber materials. Improving the photosensitive characteristics in core layer of optical fibers can markedly amplify the refractive index within the structure. Means for improving the photosensitivity are listed below:

1. Add photosensitive materials, such as Ge, Si, and Pb, to the optical fiber.
2. Expose the optical fiber to an environment filled with high-pressure hydrogen gas.
3. Bake the photosensitive area of the optical fiber repeatedly by using an intense hydrogen flame.

Two common techniques for manufacturing FBG and LPG are the phase mask method and the point-by-point (PbP) inscription method. The phase mask method uses electron-beam lithography to etch patterns onto a quartz glass substrate to manufacture a diffractive phase mask. Subsequently, a bare fiber (*i.e*., a photosensitive optical fiber) is placed on the phase mask to form FBGs under UV irradiation. The phase mask method enables the self-design of appropriate phase mask patterns to complete FBGs with different grating period (Λ*_Bragg_*). The phase mask method is advantageous because it has low light source coherence requirements during manufacturing. Once the phase mask manufacturing process is complete, mass production of phase masks can be performed with identical specifications. PbP inscription involves using high-precision devices to control grating displacement and periodically expose fiber grating during lithography. Users can control the pulling speed of the optical fibers during fabrication in order to fabricate fiber gratings of arbitrary period lengths. The PbP method is advantageous because it is highly flexible and allows arbitrary designs on the cross-section of fiber gratings. However, it also has the disadvantage of requiring complex centered optical systems and precise moving techniques if CO_2_ or femtosecond lasers are used [7,8]. In fact, it depends on the technique used. This is not true if the electric-arc or the UV lasers are used [9,10]. The mathematical equation of FBG can be expressed as follows [6]:

(1) $$n_{1}(z)=n_{1}+n_{1}\Delta \delta \;\{1+\cos(2\pi /\Lambda_{Bragg}z)\}$$

where *n*_1_ is the refractive index of the core layer, Δδ is the UV-induced refractive index variation, $n_{1}\Delta \delta$ is the peak-induced index change, and Λ*_Bragg_* is the period of FBG.

The composition of superstructure fiber Bragg gratings (SFBGs) is similar to that of regular FBGs. The major difference between the two is that the distribution of SFBGs enables on–off functions (*i.e*., duty cycles less than 100%) [11,12,13]. SFBGs with different duty cycles have different spectral characteristics such as bandwidth, number of channels, and band gap size. In other words, designers can adjust the duty cycle to achieve the optimal spectral efficiency according to the specifications of optical fiber systems [14,15]. To date, all studies on optical fiber gratings have reported that coupled-mode theory is essential in designing FBGs. However, coupled-mode theory features sophisticated, difficult-to-learn physical concepts and mathematical techniques for people who are new to the field of optical fiber grating design and only need to know the basic application aspects of this technology [16,17]. Therefore, the present study adopted a numerical simulation method to elaborate and analyze SFBGs with duty cycles of 50%, 33.33%, 14.28%, 12.5%, and 10%. A full-graphic method was adopted to demonstrate the relevance between design period and spectral characteristics of SFBGs of different duty cycles. This method lowers knowledge of the mathematics and physical concepts related to this field [18]. This study incorporated a perfectly matched layer (PML), perfectly reflecting boundary (PRB), the object-meshing method (OMM), and boundary meshing method (BMM) into the finite element method (FEM) and eigenmode expansion method (EEM) [19,20]. The simulation results include the 2D and 3D power distribution of guided modes, dispersion relation of guided modes, orthogonality relationship between modes, power loss, SFBG period Λ*_Bragg_* scanning pattern, SFBG wavelength *λ_Bragg_* scanning pattern, and power propagation of HE_11_. In other words, designers can easily and flexibly design optical fiber communication systems conforming to spectral characteristic requirements based on the simulation data reported in this article.

## 2. Superstructure Fiber Bragg Grating

**Figure 1:**
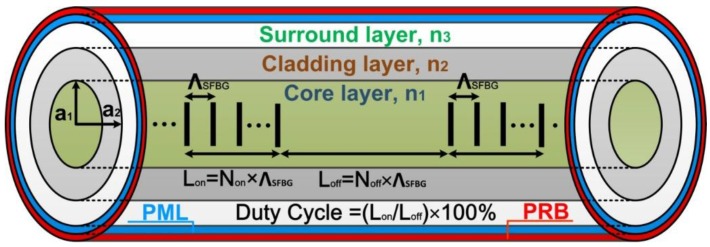
Three-dimensional geometric structure of an SFBG.

The 3D geometric structure of an SFBG is shown in Figure 1, where the on–off distribution of the fiber grating is inside the core layer; Λ*_Bragg_* is the FBG period and N_on_ and N_off_ respectively represent the number of FBGs in the on and off statuses. The unit length of FBGs in the on and off statuses are denoted as L_on_ and L_off_, respectively; the ratio of L_on_ and L_off_ is the duty cycle; and the overall on–off quantity is N. In detail, this study explored and analyzed SFBGs with five duty cycles: 50%, 33.33%, 14.28%, 12.5%, and 10%. In Figure 1, the blue area is a PML and the red area is a PRB. 

**Figure 2:**
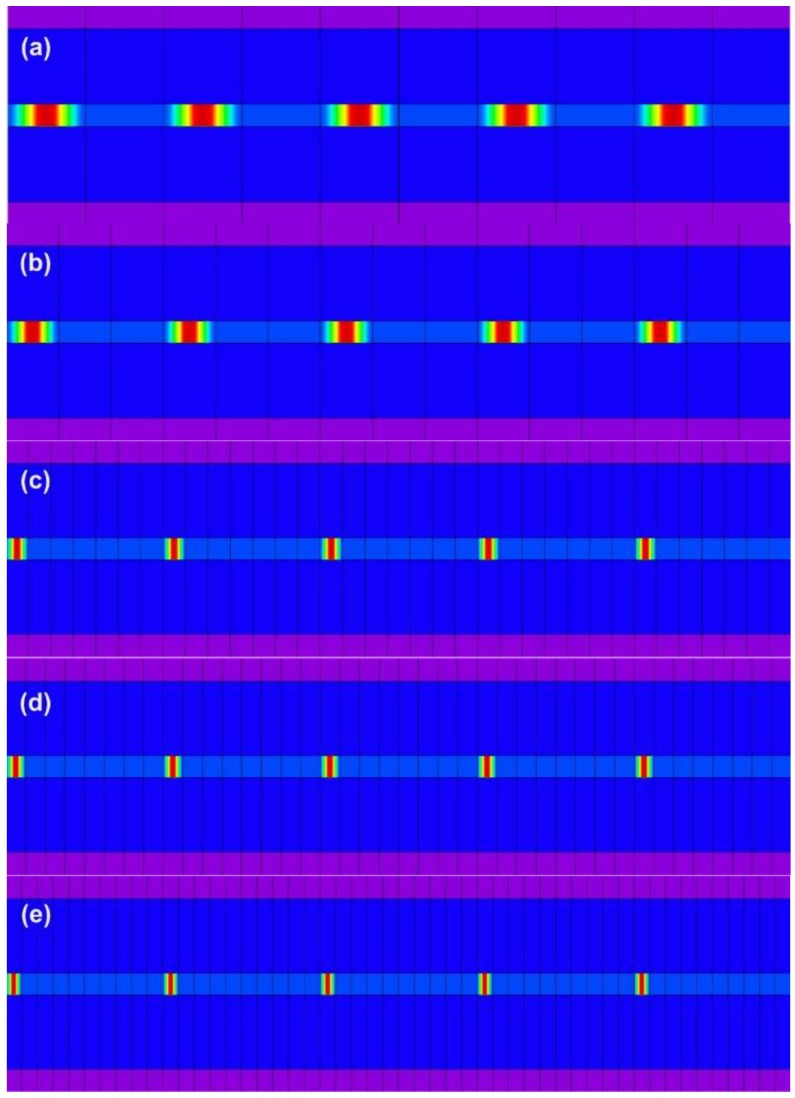
SFBG design patterns for numerical simulation and computation. (**a**) duty cycle = 50%; (**b**) duty cycle = 33.33%; (**c**) duty cycle = 14.28%; (**d**) duty cycle = 12.5%; (**e**) duty cycle = 10%.

The remaining geometric parameters and material parameters are as follows: λ = 1550 nm; a_1_ = 2.25 μm; a_2_ = 62.5 μm; n_1_ = 1.454; n_2_ = 1.43; n_3_ = 1; $n_{1}\Delta \delta =0.001$; Λ_SFBG_ = 0.5371029 μm; N_on_ = 374; N_off_ = 9× N_on_; and N = 50. The overall SFBG length is 100438.24 μm (N × Λ_SFBG_ × (N_on_ + N_off_) = 100438.24 μm). The numerical computation of the SFBGs with the five duty cycles is depicted in Figure 2. 

## 3. Finite Element Method

The FEM is a numerical simulation method involving a partial differential equation with boundary conditions. Given the rapid development of the computer industry, the FEM is an indispensable computation tool for solving engineering problems in various fields including electromagnetism, heat transfer, fluid dynamics, and structural mechanics. This numerical simulation method was established on the basis of variational principles, domain meshing, and interpolation function [21,22,23]. In the present study, FEM was primarily used to solve all possible guided modes on the SFBG X–Y plane (*i.e*., cross-section) with given boundary conditions. Mode expansion theory posits that any guided mode structure requires the concurrent existence of discrete guided modes and continuous radiation modes. However, limited by server memory capacity and calculation speed, numerical simulations involving continuous radiation modes are unlikely to be practical. To solve this problem and reduce the difference between simulation results and actual manufacturing outcomes, the present study adopted a previously proposed innovative technique to integrate PML and PRB into the FEM [19,24,25,26]. PRB converts continuous radiation modes reflected onto the boundaries into discrete guided modes. Because of the artificial material PML, the converted discrete guided modes show power loss during transmission to achieve the goal of realizing enclosed simulation environments in open and real environments. The SFBG simulation environment is depicted in Figure 1, where the blue area is the PML and the red area is the PRB. 

Despite the advantages, the fully developed FEM has a major problem as it employs the uniform triangular meshing technique [27,28,29]. When FEM uniform triangular meshing is conducted on the SFBG X–Y plane, the coexistence of large and small objects in the structure results in the need for unnecessarily high memory capacity and computational resources for the large objects to produce high resolutions for small objects. Clearly, using FEM to perform numerical calculations involves meticulous deliberation on controlling the meshing resolution. To solve this problem, this study integrated OMM and BMM into the FEM [19]. OMM enables the meshing of objects at various resolutions according to the component dimensions, thereby preventing waste of excess meshing on large-size components, and BMM enables the use of delicate meshing resolutions on object boundaries to precisely determine the geometric position of object boundaries, thereby facilitating calculating accurate material parameters in simulations. Four meshing resolution categories were adopted for the SFBGs proposed in this study: BMM, small OMM, medium OMM, and large OMM. The ratio of the triangle area divided by BMM to the OMMs was 1:5:17:43. 

**Figure 3:**
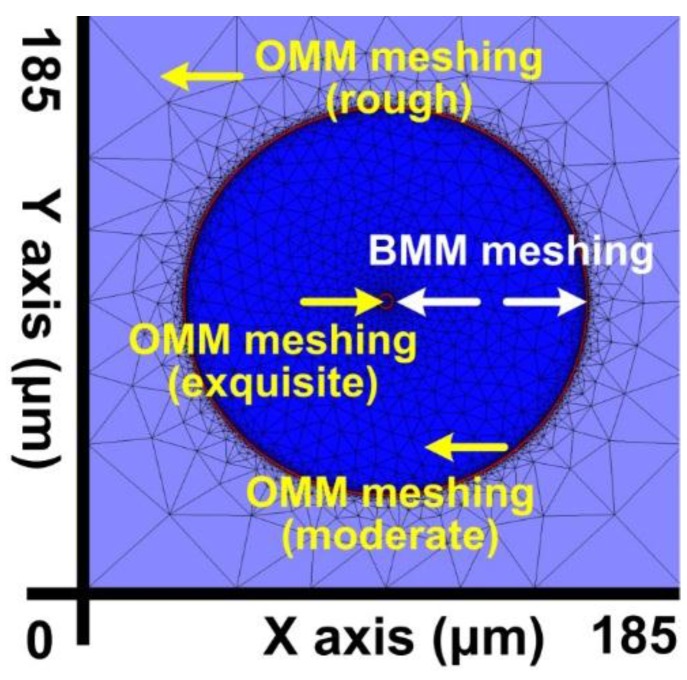
X–Y plane meshing resolution after using OMM and BMM in the FEM.

Figure 3 shows the FEM, OMM, and BMM results for the proposed SFBG geometric structure. In the figure, the different OMM meshing resolutions are relative to object size. The meshing resolutions of the core, cladding, surround layers are large, medium, and small, respectively. BMM enables finely meshed object boundaries, generating the highest meshing resolution among all objects (minimum triangle area).

From a mathematical perspective, all solutions to partial differential equations with boundary conditions must be orthogonal. In other words, the orthogonality of any two mathematical solutions must be zero, as expressed in the following equation [6].

(2) $$Orthogonality=\int_{A\infty }{\vec{E}}_{t\nu }\times {\vec{H}}_{t\mu }•\hat{z}\;dA$$

**Figure 4:**
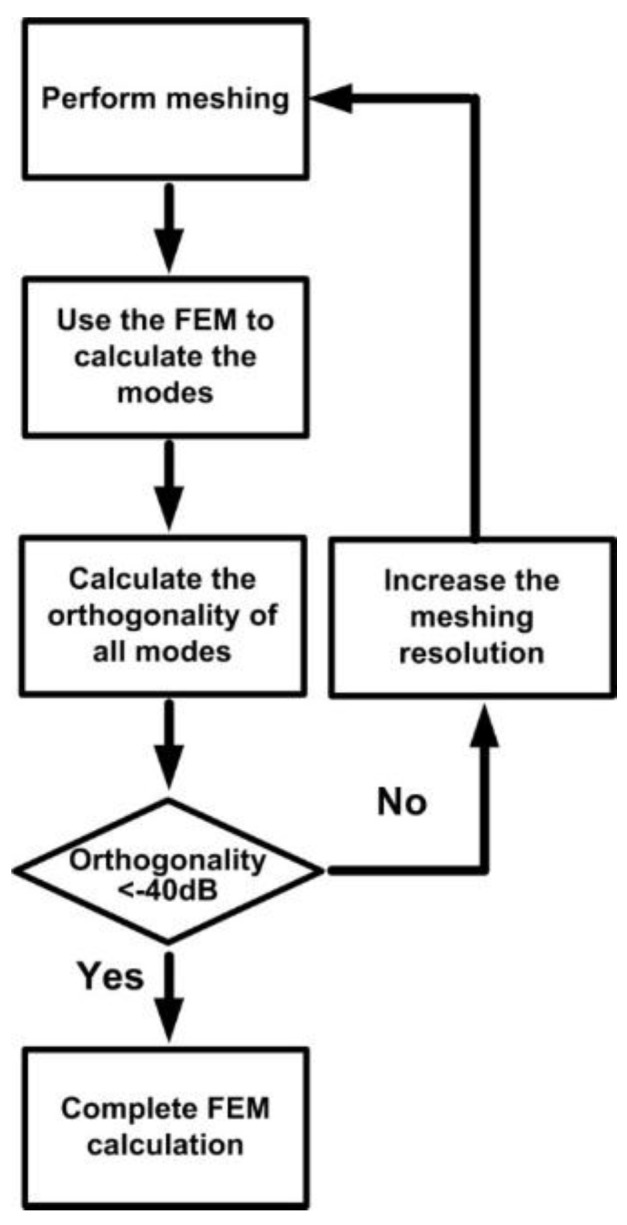
A flow diagram of error examination for FEM.

**Figure 5:**
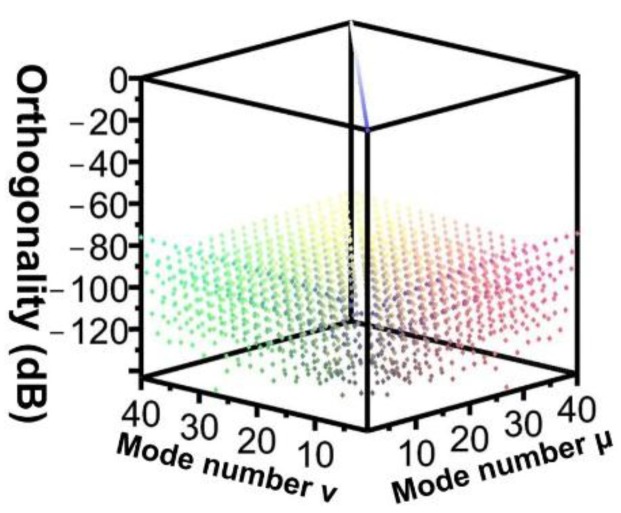
Orthogonal relationship among 40 modes.

Under limited server memory capacity and computational time, achieving complete orthogonality between modes is impossible. In other words, errors are inevitable in FEM numerical simulations. Therefore, the following cyclic error examination procedure was adopted for the FEM as shown in Figure 4. In this study, the predetermined standard for errors was set at −40 dB. Figure 5 shows the orthogonal relationship among 40 guided modes at a meshing resolution of 1:5:17:43. The results satisfy the predetermined standard of examination (*i.e*., othogonality lower than −40 dB).

## 4. Eigenmode Expansion Method

In this study, EEM enabled guided modes identified from the FEM to transmit energy in the SFBG [19,30]. First, the EEM algorithm was applied to extract and discretize the first periodic object (block) from the periodic structure of the SFBG. Meshing along the Z-axis, every meshed object was seen as a waveguide (segment) with a uniform refractive index. Subsequently, the FEM was employed to solve the modes of each segment. Through eigenmode expansion, energy conversion between the segments was performed. In accordance with the aforementioned method, sequential calculation yielded the energy transmission of the guided modes in the SFBG structure. This brief explanation of the EEM shows that because the component has a periodic structure, the guided modes in one period are identical. In other words, using the FEM was necessary only for the first block of the calculation in order to complete the solutions for the guided modes of the entire periodic object.

**Figure 6:**
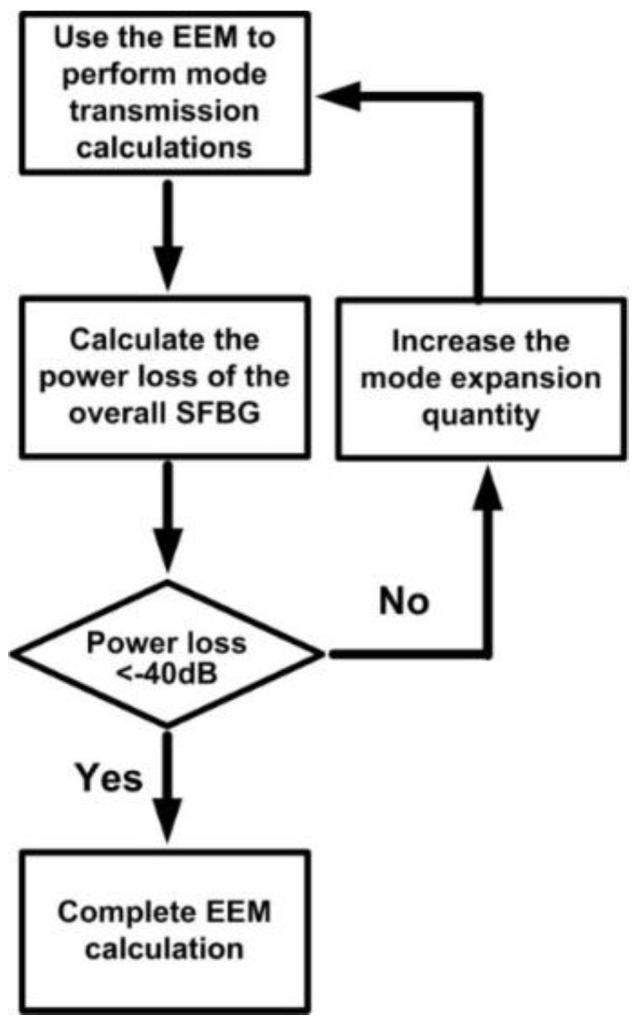
A flow diagram of error examination for EEM.

Clearly, this periodic characteristic can substantially reduce the time and memory required for conducting a numerical analysis on a whole physical mode. Therefore, integrating the FEM and EEM is suitable for performing numerical simulations on periodic structures [19]. From a pure mathematical perspective, EEM plays the role of Fourier series expansion. According to the Fourier series expansion principle, the quantity of expansion bases should be infinite. If the expansion bases are insufficient, errors are unavoidable. Because of the limited server computational power and memory capacity, conducting a numerical simulation that encompasses all bases (modes) is impossible. Therefore, a cyclic error examination procedure similar to that of the FEM was adopted for the EEM as shown in Figure 6. The predetermined standard for errors in this study was set at −40 dB.

**Figure 7:**
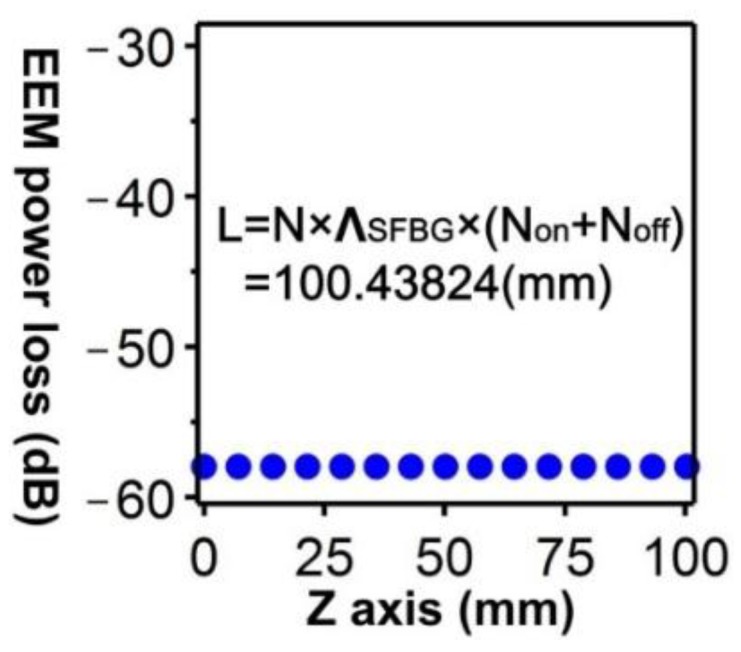
Relationship between power loss resulting from using the EEM and applying the SFBG length in the 40 guided modes.

The results of overall power losses under the 40 guided modes used in the proposed SFBG structure are depicted in Figure 7. Clearly, an SFBG with a length of 100.43824 mm satisfies the predetermined standard (lower than −40 dB). 

## 5. Design and Simulation

The numerical simulation methods and SFBG structure parameters discussed in the previous sections were applied to complete the analysis of the optical characteristics of FBGs with five duty cycles (50%, 33.33%, 14.28%, 12.5%, and 10%) through four design steps and two error examination procedures. The simulation results include the 2D and 3D power distribution of guided modes, distribution of the guided modes, orthogonality relationship among 40 modes, SFBG period Λ*_Bragg_* scanning pattern, SFBG wavelength *λ_Bragg_* scanning pattern, and power propagation pattern.

1. Use the FEM to solve the 40 guided modes on the SFBG X–Y plane.
2. Examine whether the meshing resolution error satisfies the predetermined standard (lower than −40 dB).
3. Use the EEM to propagate the power of the guided modes in the SFBG.
4. Examine whether the power loss resulting from the EEM in the 40 guided modes satisfy the predetermined standard for errors (lower than −40).
5. Calculate the relationship between the SFBG periods and power for the five duty cycles.
6. Calculate the SFBG spectra of the five duty cycles.

**Figure 8:**
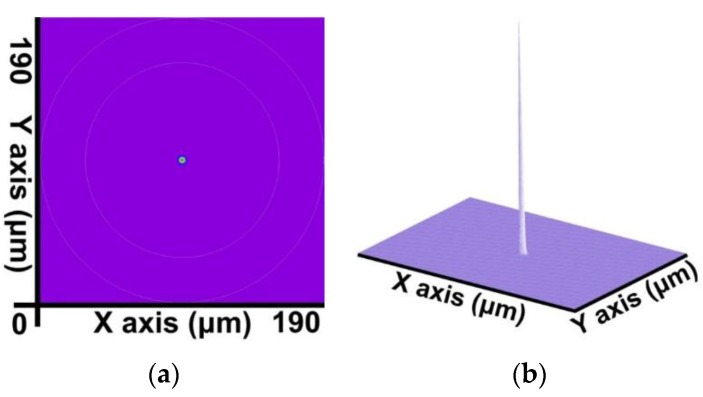
The 2D and 3D power distribution patterns of the core mode (HE_11_) with an effective refractive index of $n_{eff}^{core}=1.444086282$. (**a**) The 2D power distribution; (**b**) the 3D power distribution.

**Figure 9:**
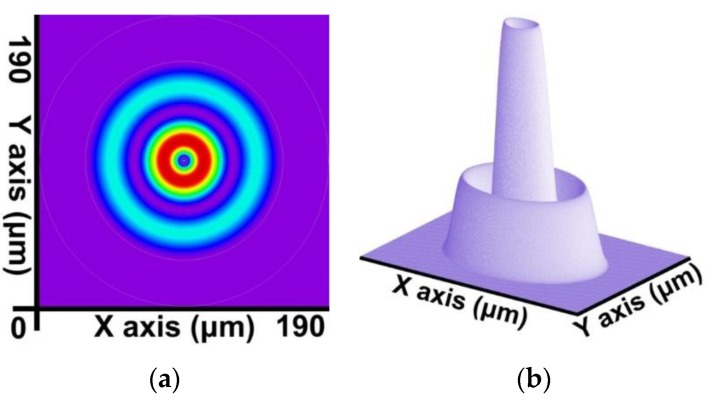
The 2D and 3D power distribution patterns of the cladding modes (v = 9) with an effective refractive index of $n_{eff}^{\nu =9}=1.429839007$. (**a**) The 2D power distribution; (**b**) the 3D power distribution.

**Figure 10:**
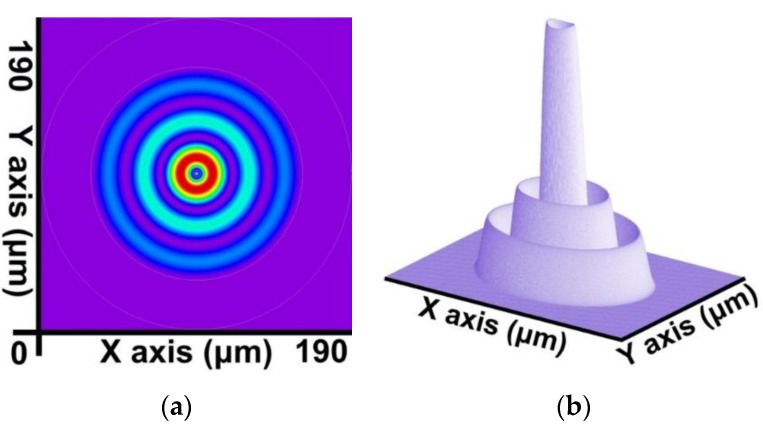
The 2D and 3D power distribution patterns of the cladding modes (v = 20) with an effective refractive index of $n_{eff}^{\nu =20}=1.429626044$. (**a**) 2D power distribution; (**b**) 3D power distribution.

**Figure 11:**
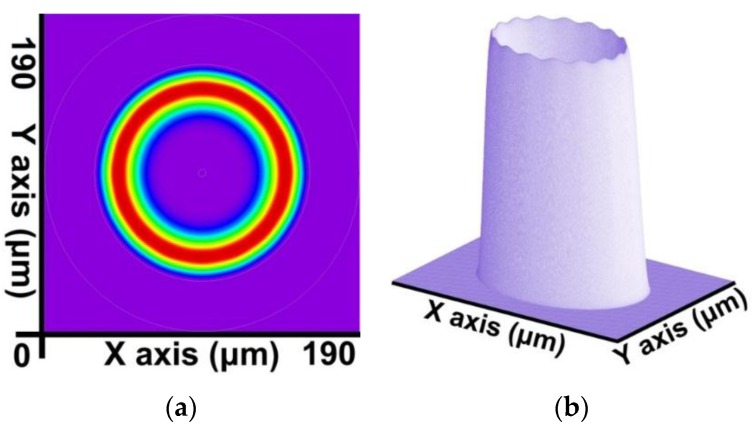
The 2D and 3D power distribution patterns of the cladding modes (v = 30) with an effective refractive index of $n_{eff}^{\nu =30}=1.429493534$. (**a**) The 2D power distribution; (**b**) the 3D power distribution.

**Figure 12:**
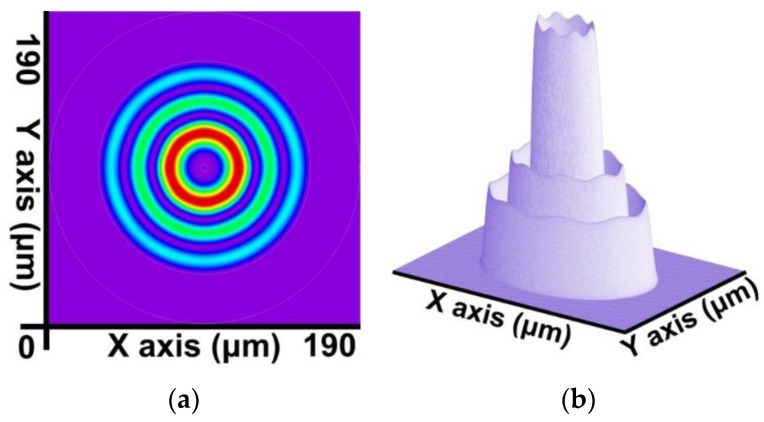
The 2D and 3D power distribution patterns of the cladding modes (v = 39) with an effective refractive index of $n_{eff}^{\nu =39}=1.429301924$. (**a**) The 2D power distribution; (**b**) the 3D power distribution.

By combining the SFBG geometric structure and parameter setting with the preceding calculation steps, a core mode (HE_11_) and 39 cladding modes were obtained. The 2D and 3D power distribution patterns of the core mode (HE_11_) are shown in Figure 8a,b, respectively; the effective refractive index is $n_{eff}^{core}=1.444086282$. The 2D and 3D power distribution patterns of the cladding modes (v = 9) are shown in Figure 9a,b; the effective refractive index is $n_{eff}^{\nu =9}=1.429839007$. The 2D and 3D power distribution patterns of the cladding modes (v = 20) are shown in Figure 10a,b, respectively; the effective refractive index is $n_{eff}^{\nu =20}=1.429626044$. The 2D and 3D power distribution patterns of the cladding modes (v = 30) are shown in Figure 11a,b, respectively; the effective refractive index is $n_{eff}^{\nu =30}=1.429493534$. The 2D and 3D power distribution patterns of the cladding modes (v = 39) are shown in Figure 12a,b, respectively; the effective refractive index is $n_{eff}^{\nu =39}=1.429301924$.

**Figure 13:**
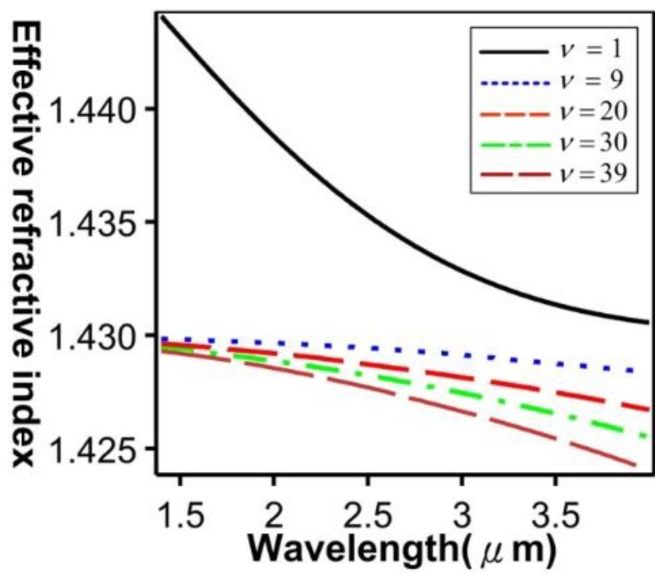
Relationship between the effective refractive indices and wavelengths of the guided modes (ν = 1, ν = 9, ν = 20, ν = 30, and ν = 39).

**Figure 14:**
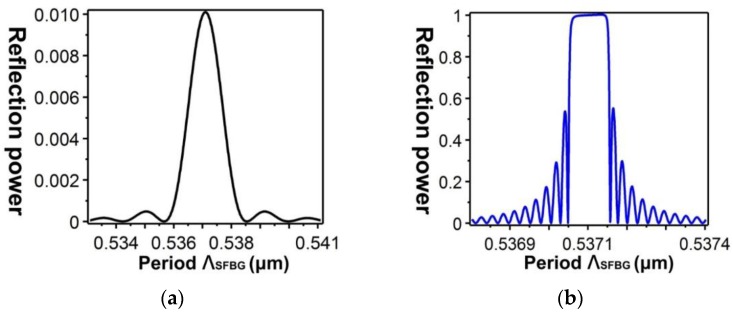
Relationship between the SFBG period and reflection power: (**a**) single SFBG; (**b**) compressed SFBG.

**Figure 15:**
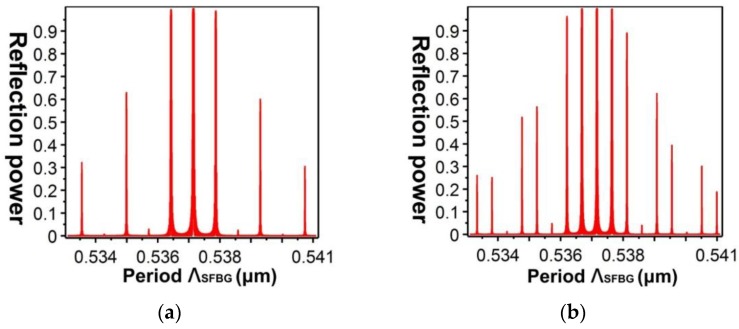

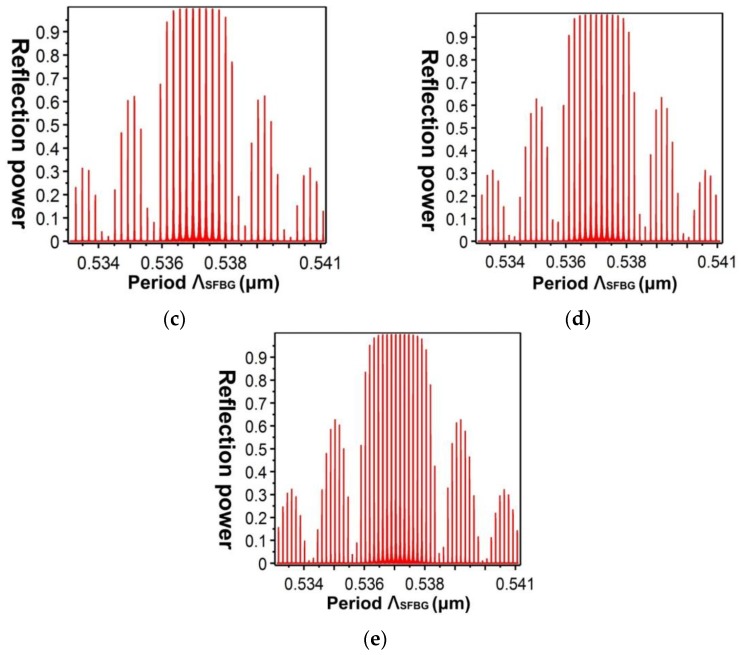
Relationship between the SFBG period and reflection power: (**a**) duty cycle = 50%; (**b**) duty cycle = 33.33%; (**c**) duty cycle = 14.28%; (**d**) duty cycle = 12.5%; (**e**) duty cycle = 10%.

**Figure 16:**
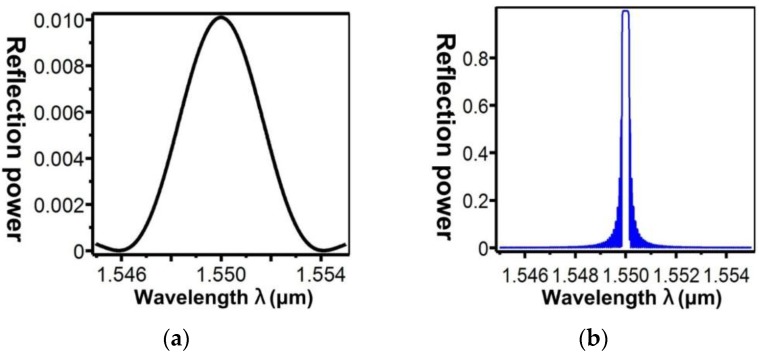
SFBG spectra at Λ_SFBG_ = 0.5371029 μm: (**a**) single SFBG; (**b**) compressed SFBG.

**Figure 17:**
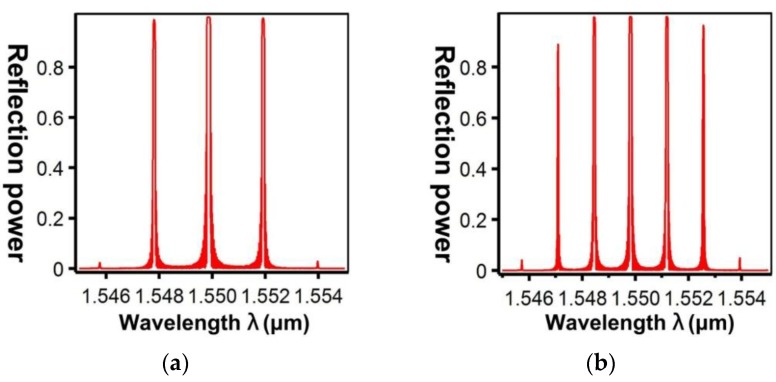

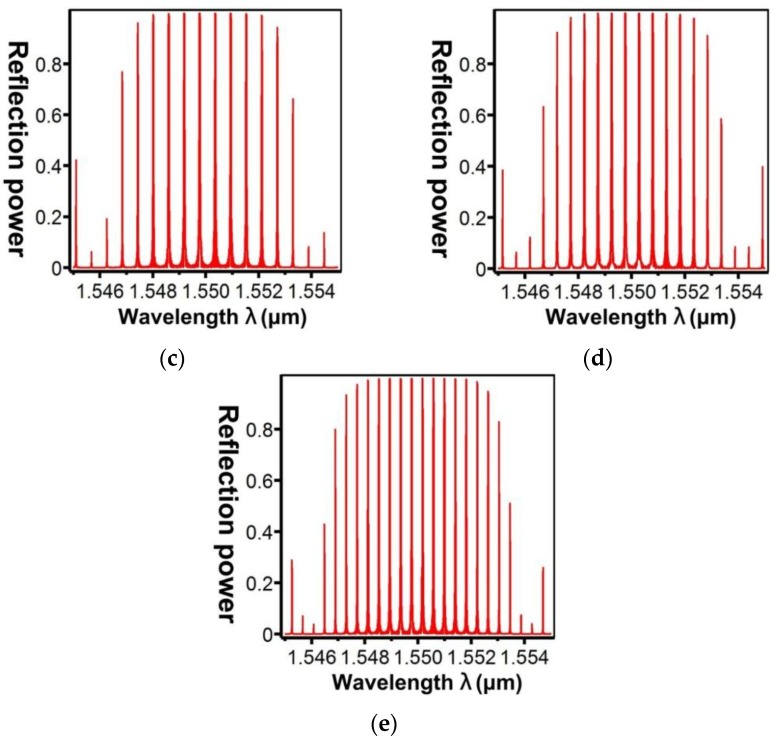
SFBG spectra at Λ_SFBG_ = 0.5371029 μm: (**a**) duty cycle = 50%; (**b**) duty cycle = 33.33%; (**c**) duty cycle = 14.28%; (**d**) duty cycle = 12.5%; (**e**) duty cycle = 10%.

Figure 13 shows the relationship between the effective refractive indices and the wavelengths of the guided modes with mode order (v) of 1, ν = 9, ν = 20, ν = 30, and ν = 39, respectively. When the mode order increases, the sensitivity to wavelength changes improves. By combining the numerical simulation methods of the FEM and EEM, the relationship between a single SFBG period and the reflection power were determined (Figure 14). The sampled grating is defined as compressed SFBG if the unmodulated regions are removed, but the modulated portion of the grating is maintained. The simulated SFBG calculations based on the five duty cycles shows the relationship between the gating period and reflection power (Figure 15). Regarding the relationship between the grating period and reflection power, Figure 15a depicts the relationship for the 50% duty cycle, Figure 15b depicts that for the 33.33% duty cycle, Figure 15c depicts that for the 14.28% duty cycle, Figure 15d depicts that for the 12.5% duty cycle, and Figure 15e depicts that for the 10% duty cycle. According to Figure 14a, the optimal design period for SFBG is Λ_SFBG_ = 0.5371029 μm. Under this period, the calculation results of a single SFBG and the compressed SFBG spectra are shown in Figure 16, and the SFBG spectra for the five duty cycles are depicted in Figure 17. When the duty cycle decreases, the bandwidth and gap band decrease whereas the number of channels increases. In other words, through the research results of the present study, designers can adjust the duty cycle to reach the optimal spectral utilization efficiency according to the specifications of an optical fiber system [31,32]. Finally, the accuracy of the analysis results was verified. 

**Figure 18:**
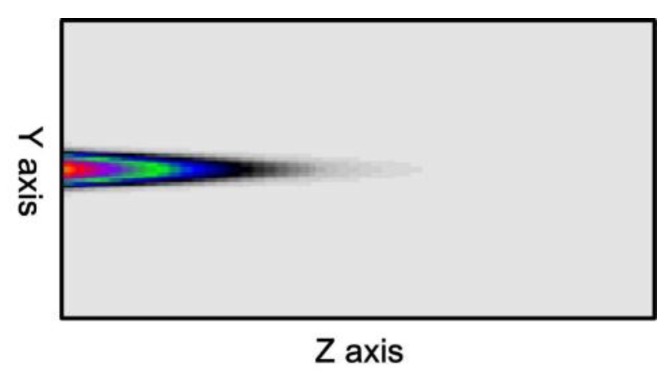
Core mode power propagation pattern on the Y–Z plane under the conditions of Λ_SFBG_ = 0.5371029 μm and duty cycle = 10%.

For example, under the 10% duty cycle, the core mode (HE_11_) shown in Figure 8 was input from the left end of Figure 1 to calculate the power propagation of SFBG. The results are shown in Figure 18. The core mode is completely reflected to the input end because of the SFBG disturbance effect during the propagation process.

## 6. Conclusions

Compared with conventional coupled-mode theory, which is complex and difficult to learn, the proposed method incorporates a numerical simulation to investigate and gain insight into SFBGs of five duty cycles by using the full-graphics method. The numerical simulation methods comprise the FEM, EEM, PML, PRB, OMM, and BMM. The modified FEM technique employed in this study can substantially improve the overall numerical calculation performance and accuracy, and reduced the gap between the simulation results and actual implementation. In addition, analysis of multiple optical characteristics of the SFBG yielded the 2D and 3D power distribution of the guided modes, dispersion relation of guided modes, orthogonality relationship between the modes, power loss, SFBG period Λ*_Bragg_* scanning pattern, SFBG wavelength λ*_Bragg_* scanning pattern, and power propagation of HE_11_. The simulation results reveal the spectral characteristics of the SFBGs of varying duty cycles (50%, 33.33%, 14.28%, 12.5%, and 10%). As the duty cycle decreases, the bandwidth and gap band decrease and the number of channels increases. In other words, designers can use the simulation data from this study to adjust the duty cycle values according to the specifications of their optical fiber systems to develop communication devices that optimize the spectral utilization efficiency.
